# Optimization of individualized therapy with oral valproic acid in pediatric patients with epilepsy based on factors influencing blood concentrations

**DOI:** 10.3389/fphar.2026.1793966

**Published:** 2026-05-04

**Authors:** Yinhui Qin, Na Zhang, Panpan Zhang, Yuetai Teng, Zhengchao Xia, Zhenkun Mao, Lei Wang, Yan Yang, Weihong Niu

**Affiliations:** 1 Department of Pharmacy, Henan Provincial People’s Hospital, Zhengzhou University People’s Hospital, Henan University People’s Hospital, Zhengzhou, Henan, China; 2 Shandong Academy of Chinese Medicine, Jinan, China; 3 School of Medicine, Henan Engineering Research Center of Funiu Mountain’s Medicinal Resources Utilization and Molecular Medicine, Pingdingshan University, Pingdingshan, China; 4 Department of Pharmacy, Jinan Vocational College of Nursing, Jinan, China; 5 Department of Pathology, Henan Provincial People’s Hospital, Zhengzhou University People’s Hospital, Henan University People’s Hospital, Zhengzhou, Henan, China

**Keywords:** blood concentration, epilepsy, individualized dosing, therapeutic drug monitoring, valproic acid

## Abstract

**Background:**

Valproic acid (VPA) is widely used in pediatric epilepsy, but its blood concentrations vary considerably among pediatric patients with epilepsy. This study aimed to investigate the factors influencing VPA blood concentrations in this population.

**Methods:**

This study included patients with epilepsy aged 0–18 years who were treated with VPA. Clinical data collected included age, gender, daily dose, biochemical parameters (liver and renal function), and electrolyte levels. VPA blood concentrations were measured by immunoassay. Correlation analyses and linear regressions were conducted to assess associations between clinical variables and VPA blood concentrations.

**Results:**

The mean VPA blood concentration was 69.82 ± 25.22 μg/mL, and 70.06% of patients had blood concentrations within the therapeutic range. Daily dose was positively correlated with blood concentration (*r* = 0.319, *P* < 0.001). Age was significantly associated with VPA blood concentration, with younger children (<6 years) receiving lower daily doses and exhibiting lower blood concentrations than older age groups (both *P* < 0.001). Creatinine and globulin levels were positively correlated with VPA blood concentrations (all *P* < 0.001), whereas alanine aminotransferase levels showed a negative correlation with blood concentrations (*P* < 0.001). Notably, calcium and phosphorus levels were negatively correlated with VPA blood concentrations.

**Conclusion:**

VPA blood concentrations in the study population were influenced by multiple factors, including daily dose, age, renal and hepatic function, and electrolyte status. Given the marked inter-individual variability, individualized treatment guided by therapeutic drug monitoring (TDM) is essential for optimizing dosing accuracy and therapeutic efficacy.

## 1 Introduction

Epilepsy is a chronic neurological disorder characterized by recurrent, unprovoked seizures resulting from excessive and abnormal neuronal activity in the brain (Fisher et al., 2014). Epilepsy affects approximately 51.7 million people worldwide and imposes a substantial burden on quality of life (Feigin et al., 2025). In pediatric populations, epilepsy commonly presents as childhood absence epilepsy, juvenile myoclonic epilepsy, generalized tonic–clonic seizures, and developmental and epileptic encephalopathies. Antiepileptic drugs remain the cornerstone of epilepsy management, aiming to suppress seizure activity by modulating neuronal excitability and synaptic transmission (Rogawski and Löscher, 2004; Li et al., 2025). Among the available antiepileptic drugs, valproic acid (VPA) is recognized as a broad-spectrum agent and remains a first-line therapy for many generalized epilepsies in children, particularly absence, myoclonic, and generalized tonic–clonic seizures. Owing to its broad efficacy across seizure types, VPA plays a pivotal role in the management of pediatric epilepsy. Its clinical efficacy is largely mediated through enhancement of γ-aminobutyric acid (GABA)–dependent inhibitory neurotransmission. This effect is achieved by increasing cerebral GABA concentrations *via* stimulation of synthesis and inhibition of degradation, leading to reduced neuronal excitability (Johannessen, 2000; Löscher, 2002; Perucca, 2002).

In clinical practice, VPA exhibits favorable pharmacokinetic properties. It is commonly administered in oral formulations, including valproate tablets and oral solutions. Following oral administration, VPA is rapidly and almost completely absorbed, with a bioavailability approaching 100%. The time to peak plasma concentration typically ranges from 1 to 4 h for immediate-release formulations, while extended-release forms exhibit delayed absorption (Perucca, 2002). In circulation, approximately 90% of VPA binds to plasma proteins, primarily albumin, and only the unbound fraction exerts pharmacological activity. The volume of distribution ranges from 0.1 to 0.4 L/kg. VPA readily crosses the blood-brain barrier, and its concentration in cerebrospinal fluid is about 10% of the corresponding plasma level (Zaccara et al., 1988). VPA is extensively metabolized in the liver, accounting for over 90% of its elimination, through three major pathways. The predominant route is glucuronidation (approximately 30%–50%), mediated by uridine 5′-diphospho-glucuronosyltransferase (UGT) enzymes such as UGT1A6, UGT1A9, and UGT2B7, resulting in pharmacologically inactive VPA-glucuronide conjugates excreted in urine. The second pathway involves mitochondrial β-oxidation (30%–40%), analogous to fatty acid metabolism, generating intermediates such as 3-oxo-VPA and 2-ene-VPA (Silva et al., 2008). Notably, this pathway also produces potentially hepatotoxic metabolites, including 4-ene-VPA. A minor portion (∼10%) undergoes cytochrome P450 (CYP)-mediated oxidation, involving isoenzymes such as CYP2C9, CYP2A6, and CYP2B6, leading to the formation of reactive metabolites like 4-ene-VPA and E-2,4-diene-VPA, which have been implicated in hepatotoxicity (Zhu et al., 2017). Ultimately, more than 90% of VPA and its metabolites are eliminated *via* the kidneys, with less than 3% excreted unchanged (Gugler and von Unruh, 1980). The elimination half-life varies with age, ranging from approximately 6–12 h in children to 9–16 h in adults.

In pediatric practice, maintaining VPA blood concentrations within the therapeutic range (50–100 μg/mL) increases the likelihood of seizure control while limiting concentration-related adverse effects. However, despite this clinical anchor, findings from prior pediatric studies are difficult to generalize to bedside decision-making. Many cohorts were small and accrued over short periods, limiting statistical power for age-stratified analyses and for detecting modest yet clinically meaningful effects. Numerous reports examined only one or two variables and did not perform multivariable adjustment, leaving confounding by age, organ function, and co-medications insufficiently addressed. Age categories were often broad or heterogeneous, obscuring developmental inflection points relevant to dosing (<6, 6–12, >12 years). Finally, few studies have comprehensively evaluated the effects of hepatic and renal function indicators as well as electrolyte levels on VPA blood concentrations, despite the fact that these parameters may significantly influence drug distribution, binding, and elimination (Verrotti et al., 2019). Collectively, these limitations hinder individualized VPA therapy in pediatric patients with epilepsy.

To address these gaps, we evaluated how VPA blood concentrations relate to age, gender, daily dose, hepatic and renal indicators, electrolyte levels, and commonly coadministered antiseizure medications. Analyses included correlation tests, nonparametric group comparisons, and linear regression. We hypothesized that: (i) daily dose would be positively correlated with VPA blood concentrations; (ii) children younger than 6 years would receive lower daily doses and consequently exhibit lower VPA blood concentrations; (iii) routinely available hepatic, renal, and electrolyte indicators—particularly globulin, alanine aminotransferase, creatinine, and phosphorus—would show independent associations with VPA blood concentrations. Finally, we aim to translate these findings into actionable clinical guidance. Collectively, this approach guided by therapeutic drug monitoring (TDM) is intended to shorten time to seizure control, reduce concentration-related adverse effects, avoid unnecessary dose escalation or polytherapy, and lower unplanned healthcare utilization, thereby improving the precision, safety, and effectiveness of VPA therapy in pediatric patients with epilepsy (Johannessen and Tomson, 2006; van Dijkman et al., 2018).

## 2 Materials and methods

### 2.1 General information

A total of 638 epilepsy patients aged 0–18 years who visited or were hospitalized at our institution between January 2022 and December 2023 were retrospectively enrolled in this study. According to relevant literature and national guidelines, the therapeutic range for VPA at our hospital is defined as 50–100 μg/mL (Patsalos et al., 2018; Reimers et al., 2018; Pretorius et al., 2024). Clinical data collected included gender, age, daily dose, liver and renal function indicators, electrolyte levels, and concomitant antiepileptic drug use. Inclusion criteria were as follows: (1) Diagnosis of epilepsy according to the latest International League Against Epilepsy classification, without restriction on seizure type, including generalized, focal, and developmental and epileptic encephalopathies; (2) Age between 0 and 18 years; (3) Twice-daily oral VPA therapy with TDM, administered either as monotherapy or in combination with one additional antiepileptic drug; (4) Achievement of steady-state blood concentration was defined as continuous oral administration of an unchanged VPA dose for at least 2 weeks, with consistent dosing intervals and the same formulation, to ensure attainment of steady-state levels. Only patients who met all four criteria were included in the final analysis. Exclusion criteria included: (1) Non-standardized trough sampling (blood not collected ∼30 min before the next scheduled dose); (2) Insufficient VPA treatment duration to achieve steady state; (3) Incomplete or missing essential clinical data, including daily dose records and laboratory parameters such as hepatic and renal function indicators and electrolyte levels; (4) Determined by the research team to be otherwise unsuitable for inclusion. Patients meeting one or more exclusion criteria were excluded from the study.

### 2.2 Main drugs, reagents, and equipment

The drugs and reagents used in this study included sodium valproate tablets (200 mg), magnesium valproate tablets (200 mg), sodium valproate oral solution (40 mg/mL), purified water, VPA quality control products, VPA determination kits, VPA calibration products, dilute hydrochloric acid solution, and sodium hypochlorite solution. The equipment utilized included a Viva-E fully automated biochemical analyzer, HYC-410 medical refrigerator, DW-25L262 medical low-temperature freezer, SC-3616 low-speed centrifuge, VX200-T vortex mixer, and H1650-W benchtop high-speed microcentrifuge.

### 2.3 Collection and measurement methods of blood samples

For each enrolled pediatric epilepsy patient, a peripheral venous blood sample (2–3 mL) was obtained 30 min prior to the next scheduled dose on the day of TDM. Particular attention was paid to blood sampling in infants, which was performed by experienced pediatric nurses using age-appropriate venipuncture techniques, and only the minimum volume required for analysis was collected to ensure patient safety. Each blood sample obtained from enrolled pediatric epilepsy patients was collected in an individual serum separator tube, allowed to clot at room temperature, and centrifuged at 3,500 rpm for 5 min. The serum supernatant was separated promptly, and a 150 μL aliquot was analyzed on a Viva-E biochemical analyzer using a homogeneous enzyme immunoassay to determine steady-state trough concentrations of VPA, which are hereafter referred to as VPA blood concentrations throughout the manuscript. All assays were performed according to the manufacturer’s instructions, with routine internal quality control procedures applied to ensure analytical accuracy.

### 2.4 Statistical methods

Statistical analyses were conducted using SPSS version 27. Descriptive statistics were used to summarize and characterize the clinical data. Multiple linear regression analysis was performed to evaluate the effects of liver and renal function indicators, as well as electrolyte levels, on VPA blood concentrations. Pearson correlation analysis was employed to assess the relationships between VPA blood concentration and daily dose, renal function indicators, and electrolyte levels. Simple linear regression analysis was conducted to explore the linear associations of daily dose, creatinine, calcium, and phosphate levels with VPA blood concentration. Renal function indicators, liver function indicators, and electrolyte indicators were initially categorized into three subgroups according to clinical reference ranges: low group (below normal), medium group (within the normal range), and high group (above normal). For indicators that could be classified into only two groups according to these criteria, comparisons were performed between the two groups when both groups contained more than 20 patients. For indicators that could be classified into three groups, three-group analyses were performed only when all groups contained more than 20 patients. If only two of the three groups contained more than 20 patients, analyses were restricted to those two groups. This approach was adopted to avoid unstable statistical results and unreliable inference associated with very small subgroup sizes. The Kruskal–Wallis H test was applied for comparisons among three groups, and the Mann–Whitney U test was used for pairwise comparisons between two groups. A two-tailed P-value <0.05 was considered statistically significant. Missing data were handled using a complete-case analysis approach, whereby patients with incomplete essential variables were excluded from the final analysis to maintain data integrity and analytical robustness.

## 3 Results

### 3.1 General information

A total of 638 epilepsy patients aged 0–18 years were included in the study (Table 1). The majority were outpatients (n = 521, 81.66%), with most receiving care in pediatric clinics (n = 519, 81.35%). Only a small number of patients visited neurosurgery clinics (n = 2, 0.31%). Inpatients accounted for 114 cases (17.87%), predominantly admitted to the pediatric ward (n = 110, 17.24%), followed by the neurology ward (n = 4, 0.63%). Emergency visits were rare, with only 3 patients (0.47%) seen in the pediatric emergency clinic. Regarding payment methods, the vast majority of patients (n = 623, 97.65%) were self-funded. Only 15 patients were covered by national medical insurance, including 9 from other provinces (1.41%) and 6 from local city residents (0.94%).

**TABLE 1:** Distribution of visit types and payment categories for the study subjects.

Patient information (N = 638)		Cost information (N = 638)	
Patient type	n (%)	Payment category	n (%)
Outpatient	521 (81.66)	National medical insurance (residents from other provinces)	9 (1.41)
Pediatric clinic	519 (81.35)		
Neurosurgery clinic	2 (0.31)		
Inpatient	114 (17.87)	National medical insurance (for city residents)	6 (0.94)
Pediatric ward	110 (17.24)		
Neurology ward	4 (0.63)		
Emergency patient	3 (0.47)	Self-funded	623 (97.65)
Pediatric emergency clinic	3 (0.47)		

Table 2 presents the general demographic and clinical information of the study population. This study included 480 males (75.24%) and 158 females (24.76%). The age distribution was as follows: 6–12 years (n = 307, 48.12%), >12 years (n = 216, 33.86%), and <6 years (n = 115, 18.03%). The mean ± SD of daily dose was 633.13 ± 246.33 mg/d. The mean ± SD of VPA blood concentration was 69.82 ± 25.22 μg/mL, falling within the recommended therapeutic range (50–100 μg/mL). Renal function parameters, including creatinine (46.96 ± 11.24 μmol/L), uric acid (320.61 ± 80.56 μmol/L), urea (4.77 ± 1.21 mmol/L), retinol-binding protein (32.94 ± 7.22 mg/L), and cystatin C (0.81 ± 0.09 mg/L), were all within normal reference intervals. Liver function indicators showed that the mean ± SD of alanine aminotransferase (14.11 ± 11.91 U/L), aspartate aminotransferase (24.56 ± 9.69 U/L), albumin (44.73 ± 2.77 g/L), globulin (25.01 ± 3.78 g/L), total bilirubin (7.56 ± 3.27 μmol/L), and glutamyl transpeptidase (18.61 ± 9.45 U/L) were all within clinically accepted normal ranges. However, the mean ± SD of alkaline phosphatase level was 214.85 ± 75.27 U/L, which exceeded the normal upper limit (45–125 U/L). Electrolyte levels were also within expected physiological ranges: potassium (4.48 ± 0.33 mmol/L), sodium (139.86 ± 2.49 mmol/L), chloride (105.90 ± 2.22 mmol/L), and calcium (2.48 ± 0.08 mmol/L). But the mean ± SD of phosphorus level was 1.51 ± 0.18 mmol/L, located at the upper limit of the normal reference range (0.85–1.51 mmol/L).

**TABLE 2:** The distribution of gender, age, medication dosage, blood concentrations, liver and kidney function, and electrolyte levels for the study subjects.

Characteristic	Distribution (N = 638)	Characteristic	Distribution (N = 638)
**Age**	​	**Gender**	​
<6 years, n (%)	115 (18.03)	Male, n (%)	480 (75.24)
6–12 years, n (%)	307 (48.12)	Female, n (%)	158 (24.76)
>12 years, n (%)	216 (33.86)	**Renal function indicators** ^a^	​
**Common indicators** ^a^	​	Creatinine (μmol/L)	46.96 ± 11.24
Daily dose (mg/d)	633.13 ± 246.33	Uric acid (μmol/L)	320.61 ± 80.56
Blood concentration (μg/mL)	69.82 ± 25.22	Urea (mmol/L)	4.77 ± 1.21
**Liver function indicators** ^a^	​	Retinol-binding protein (mg/L)	32.94 ± 7.22
Alanine aminotransferase (U/L)	14.11 ± 11.91	Cystatin C (mg/L)	0.81 ± 0.09
Aspartate aminotransferase (U/L)	24.56 ± 9.69	**Electrolyte levels** ^a^	​
Albumin (g/L)	44.73 ± 2.77	Potassium (mmol/L)	4.48 ± 0.33
Globulin (g/L)	25.01 ± 3.78	Sodium (mmol/L)	139.86 ± 2.49
Total bilirubin (μmol/L)	7.56 ± 3.27	Chlorine (mmol/L)	105.90 ± 2.22
Alkaline phosphatase (U/L)	214.85 ± 75.27	Calcium (mmol/L)	2.48 ± 0.08
Glutamyl transpeptidase (U/L)	18.61 ± 9.45	Phosphorus (mmol/L)	1.51 ± 0.18

^a^ Distributed data was expressed using the mean ± standard deviation (SD).

### 3.2 Correlation between daily dose and VPA blood concentration

#### 3.2.1 Study on the linear relationship between daily dose and VPA blood concentration

**FIGURE 1:**
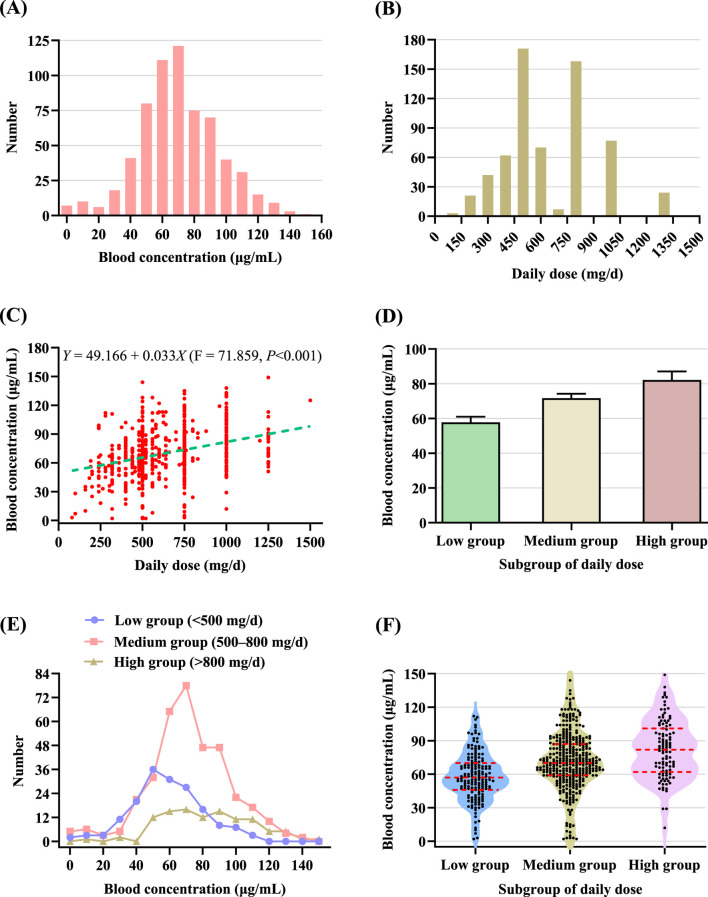
Distribution blood concentrations across different daily dose subgroups. **(A)** Frequency distribution of blood concentrations. **(B)** Frequency distribution of daily dose. **(C)** Scatter plot of the relationship between daily dose and blood concentration, with a fitted linear regression line (green dashed line). Each red dot represents an individual patient’s data point. **(D)** Mean blood concentrations across the three daily dose subgroups, with error bars indicating the 95% CI. **(E)** Frequency distribution of blood concentrations across three subgroups based on daily dose. **(F)** Violin plot of blood concentration for the three subgroups of daily dose, with red dashed lines representing the Q1, median, and Q3, respectively.

Figure 1A presents the distribution of VPA blood concentrations. Values ranged from 0 μg/mL to 150 μg/mL, with a concentration peak between 60 and 70 μg/mL. Although most values fell within the therapeutic range of 50–100 μg/mL, a notable proportion of patients still exhibited subtherapeutic or supratherapeutic levels. Figure 1B illustrates the distribution of daily dose. The administered doses ranged from below 300 mg/d to over 1,200 mg/d, with a notable clustering between 450 and 900 mg/d. The most frequently prescribed doses were approximately 500 and 800 mg/d. The Pearson correlation coefficient (*r* = 0.319, *P* < 0.001) indicates a statistically significant and modest positive association between daily dose and blood concentration. Figure 1C presents the regression analysis between daily dose and blood concentration. The fitted regression line demonstrates a positive linear trend, with equation *Y* = 49.166 + 0.033*X*, where *Y* represents the blood concentration and *X* represents the daily dose. This model suggests that for every 1 mg/d increase in VPA dosage, the corresponding blood concentration rises by approximately 0.033 μg/mL on average. Despite the significance of the trend, the broad dispersion of data points around the regression line highlights substantial inter-individual variability.

#### 3.2.2 Grouping study based on daily dose

Table 3 summarizes the distribution of VPA blood concentrations stratified by daily dose in the study population. For the subgroup of daily dose, the low group (<500 mg/d) had a mean blood concentration of 57.78 μg/mL (95% CI: 54.55–61.01), which was significantly lower than that of the medium group (500–800 mg/d) (71.71 μg/mL, 95% CI: 69.18–74.24) and the high group (>800 mg/d) (82.16 μg/mL, 95% CI: 77.20–87.12). Additionally, the mean blood concentration in the medium group was significantly lower than that in the high group (Figure 1D). A significant overall difference among the three groups was observed using the Kruskal–Wallis H test (*P* < 0.001). Post hoc pairwise comparisons with Bonferroni correction revealed significant differences between low group and medium group (adjusted *P* < 0.001), low group and high group (adjusted *P* < 0.001), and medium group and high group (adjusted *P* = 0.004).

**TABLE 3:** Comparison of blood concentrations across subgroups categorized by daily dose.

Dose grouping	n (%)	Blood concentration (μg/mL)					
		Mean (95% CI)	Median	Q1	Q3	IQR	*P* ^a^
Low group (<500 mg/d)	167 (26.18)	57.78 (54.55–61.01)	57.00	46.00	70.00	24.00	<0.001
Medium group (500–800 mg/d)	364 (57.05)	71.71 (69.18–74.24)	70.00	59.00	87.00	28.00	
High group (>800 mg/d)	107 (16.77)	82.16 (77.20–87.12)	82.00	62.00	101.00	39.00	
Low group vs. medium group	NA	NA	NA	NA	NA	NA	<0.001
Low group vs. high group	NA	NA	NA	NA	NA	NA	<0.001
Medium group vs. high group	NA	NA	NA	NA	NA	NA	0.004

Abbreviations: NA, not applicable; CI, confidence interval; Q1, first quartile; Q3, third quartile; IQR, interquartile range.

^a^ Comparisons among the three groups were conducted using the Kruskal–Wallis H test, followed by Bonferroni-corrected pairwise comparisons when appropriate.

The medium group (500–800 mg/d) had the largest number of patients and displayed a concentration peak between 60 and 80 μg/mL, with a distribution resembling a near-normal curve. The low group (<500 mg/d) exhibited a broader distribution skewed toward lower concentrations, with a peak occurring around 50 μg/mL, and a substantial proportion of patients presenting with subtherapeutic levels (<50 μg/mL). In contrast, the high group (>800 mg/d) demonstrated a flatter and more dispersed distribution (Figure 1E). This group exhibited considerable variability in blood concentrations, likely due to inter-individual differences in pharmacokinetics. The low group (<500 mg/d) had a median blood concentration of 57.00 μg/mL, with the first quartile (Q1) at 46.00 μg/mL, the third quartile (Q3) at 70.00 μg/mL, and an interquartile range (IQR) of 24.00 μg/mL. The medium group (500–800 mg/d) exhibited a higher median of 70.00 μg/mL (Q1: 59.00, Q3: 87.00, IQR: 28.00 μg/mL), while the high group (>800 mg/d) had the highest median blood concentration of 82.00 μg/mL (Q1: 62.00, Q3: 101.00, IQR: 39.00 μg/mL) (Figure 1F; Table 3). A clear upward trend in median blood concentrations was observed across the three subgroups of daily dose, reinforcing the positive association between daily dose and blood concentration. Furthermore, the IQR increased progressively across the three groups with rising daily doses, indicating greater variability in blood concentrations among patients receiving higher daily doses. This was particularly notable in the high-dose group, which demonstrated a broader and flatter density profile.

### 3.3 Influence of age and gender on VPA blood concentration

**FIGURE 2:**
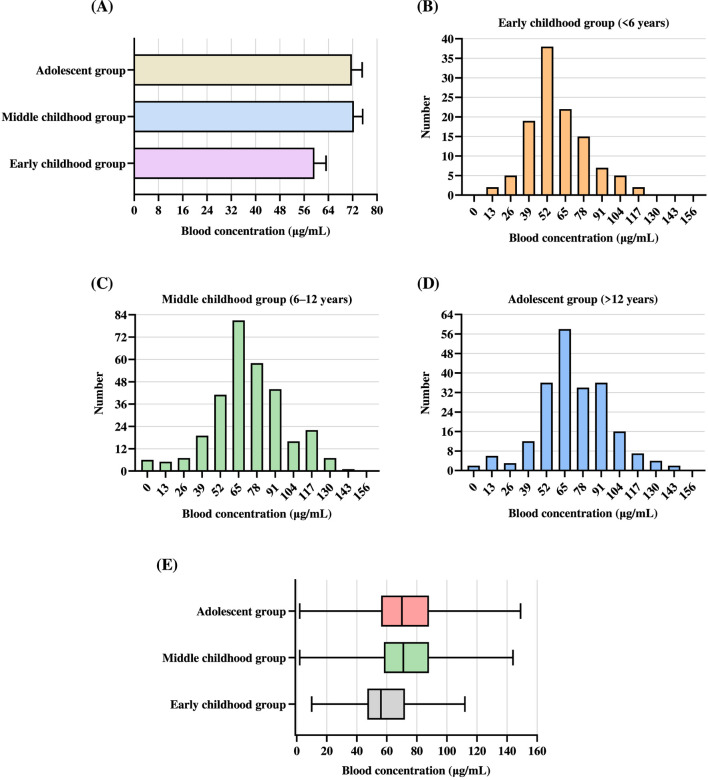
Comparison of blood concentrations across different age subgroups. **(A)** Mean blood concentrations with 95% CI across the early childhood group (<6 years), middle childhood group (6–12 years), and adolescent group (>12 years). **(B–D)** Frequency distributions of blood concentrations in the early childhood group (<6 years) **(B)**, middle childhood group (6–12 years) **(C)**, and adolescent group (>12 years) **(D)**. **(E)** Box plots of blood concentrations in the three age groups. Each box represents the IQR, with the vertical line inside the box representing the median blood concentration.

Age groups were defined according to developmental stage guidelines from the World Health Organization, and were categorized as early childhood (<6 years), middle childhood (6–12 years), and adolescence (>12 years) (Table 4). The study population did not include any newborns, and only two participants were infants (<1 year); therefore, these infants were included in the early childhood group (<6 years) due to the very small sample size. Early childhood group (<6 years) received the lowest mean daily dose (371.48 mg/d; 95% CI: 349.36–393.60), which was significantly less than that administered to middle childhood group (6–12 years) (597.11 mg/d; 95% CI: 578.43–615.79) and adolescent group (>12 years) (823.62 mg/d; 95% CI: 791.36–855.88). Notably, the mean daily dose in the middle childhood group (6–12 years) was also significantly lower than that in the adolescent group (>12 years), indicating a clear positive relationship between age and daily dose. The Kruskal–Wallis H test revealed a significant overall difference in the daily dose among the three age groups (*P* < 0.001). Post hoc pairwise comparisons with Bonferroni correction showed significant differences between each pair of groups (all adjusted *P* < 0.001). The mean blood concentration was lowest in the early childhood group (<6 years) (59.41 μg/mL; 95% CI: 55.58–63.23) and highest in the middle childhood group (6–12 years) (72.36 μg/mL; 95% CI: 69.45–75.27), followed closely by the adolescent group (>12 years) (71.74 μg/mL; 95% CI: 68.38–75.11) (Figure 2A). A Kruskal–Wallis H test showed a significant overall difference in blood concentrations among the three age groups (*P* < 0.001). Post hoc pairwise comparisons with Bonferroni correction revealed that the early childhood group (<6 years) differed significantly from the middle childhood group (6–12 years) (adjusted *P* < 0.001) and the adolescent group (>12 years) (adjusted *P* < 0.001), whereas no significant difference was observed between the middle childhood group (6–12 years) and adolescent group (>12 years) (adjusted *P* > 0.05).

**TABLE 4:** Comparison of blood concentrations across different age and gender subgroups.

Grouping	Daily dose (mg/d)		Blood concentration (µg/mL)					
	Mean (95% CI)	*P* ^a^	Mean (95% CI)	Median	Q1	Q3	IQR	*P* ^a^
**Age subgroup**	​	​	​	​	​	​	​	​
<6 years	371.48 (349.36–393.60)	<0.001	59.41 (55.58–63.23)	56.00	47.00	72.00	25.00	<0.001
6–12 years	597.11 (578.43–615.79)		72.36 (69.45–75.27)	71.00	58.00	88.00	30.00	
>12 years	823.62 (791.36–855.88)		71.74 (68.38–75.11)	70.00	56.25	88.00	31.75	
<6 years vs. 6–12 years	NA	<0.001	NA	NA	NA	NA	NA	<0.001
<6 years vs. >12 years	NA	<0.001	NA	NA	NA	NA	NA	<0.001
6–12 years vs. >12 years	NA	<0.001	NA	NA	NA	NA	NA	1.000
**Gender subgroup**	​	​	​	​	​	​	​	​
Male	655.53 (633.70–677.36)	<0.001	69.54 (67.38–71.69)	67.00	54.25	85.00	30.75	0.630
Female	565.08 (526.83–603.33)		70.67 (66.17–75.17)	69.00	53.75	89.50	35.75	

Abbreviations: NA, not applicable; CI, confidence interval; Q1, first quartile; Q3, third quartile; IQR, interquartile range.

^a^ Comparisons among the three groups were conducted using the Kruskal–Wallis H test, followed by Bonferroni-corrected pairwise comparisons when appropriate. Comparisons between two groups were performed using the Mann–Whitney U test.

In addition to age-related differences, gender-based comparisons were performed (Table 4). The mean daily dose was significantly higher in males than in females (655.53 mg/d vs. 565.08 mg/d; *P* < 0.001). However, no significant gender-related difference was observed in blood concentrations. The mean blood concentration was 69.54 μg/mL (95% CI: 67.38–71.69) in males and 70.67 μg/mL (95% CI: 66.17–75.17) in females, with no significant difference between the two groups (*P* > 0.05). Median values were also comparable (67.00 μg/mL in males vs. 69.00 μg/mL in females). In conclusion, gender had a significant influence on the daily dose but did not affect blood concentrations in this pediatric population.

Among the early childhood group (<6 years), blood concentrations ranged from 0 to 120 μg/mL, with the majority of values distributed between 39 and 78 μg/mL (Figure 2B). The most frequently observed blood concentration was approximately 52 μg/mL, representing the peak of the distribution. A secondary aggregation was noted between 65 and 78 μg/mL. In contrast, only a small number of patients had blood concentrations exceeding 100 μg/mL. Notably, a considerable proportion of patients had subtherapeutic blood concentrations (<50 μg/mL), particularly within the 0–39 μg/mL range. In the middle childhood group (6–12 years), blood concentrations ranged from 0 to 150 μg/mL, with the majority falling between 52 and 91 μg/mL (Figure 2C). The distribution demonstrated a near-normal, symmetrical pattern centered within the therapeutic range. The most common blood concentration was approximately 65 μg/mL, with the peak frequency observed at this interval (∼84 patients). Secondary peaks occurred at 78 μg/mL (∼60 patients). These findings indicate that most patients in this age group achieved VPA levels within the recommended therapeutic range of 50–100 μg/mL. In the adolescent group (>12 years), blood concentrations ranged from 0 to 150 μg/mL, with the majority of values falling between 52 and 91 μg/mL (Figure 2D). The distribution approximated a normal curve, with a slight rightward skew and moderate variability. The highest frequency was observed at 65 μg/mL, where approximately 60 patients were clustered. Secondary peaks occurred at 52, 78, and 91 μg/mL, each with 32–40 individuals. The proportion of patients with subtherapeutic blood concentrations (<50 μg/mL) was relatively low, while a subset of individuals had supratherapeutic levels exceeding 100 μg/mL. These results indicate that the majority of adolescents maintained blood concentrations within the recommended therapeutic range of 50–100 μg/mL; however, a subset of patients exceeded the upper limit of this range and warrant special attention.

The early childhood group (<6 years) had the lowest median blood concentration at 56.00 μg/mL, with the Q1 and Q3 at 47.00 μg/mL and 72.00 μg/mL, respectively, resulting in an IQR of 25.00 μg/mL. In comparison, the middle childhood group (6–12 years) exhibited a higher median blood concentration of 71.00 μg/mL (Q1: 58.00 μg/mL; Q3: 88.00 μg/mL; IQR: 30.00 μg/mL). Similarly, the adolescent group (>12 years) demonstrated a median blood concentration of 70.00 μg/mL, with the Q1 and Q3 values of 56.25 μg/mL and 88.00 μg/mL, respectively, yielding an IQR of 31.75 μg/mL (Figure 2E; Table 4). These results indicate a clear increase in median blood concentrations from early childhood group (<6 years) to older age groups. Moreover, the IQR values progressively widened with age, suggesting increased inter-individual variability in VPA blood concentrations among the middle childhood group (6–12 years) and adolescent group (>12 years). Notably, the middle childhood and adolescent groups demonstrated comparable medians and dispersion metrics (Q1, Q3, and IQR), implying a potential plateau in VPA pharmacokinetics beyond the age of 6 years.

### 3.4 Correlation between renal function indicators and VPA blood concentration

#### 3.4.1 Multiple linear regression analysis of renal function indicators and VPA blood concentration

To evaluate the influence of renal function on blood concentrations, a multiple linear regression analysis was conducted (Table 5). Creatinine demonstrated a statistically significant positive association with blood concentration (B = 0.452, β = 0.201, *P* < 0.001). In contrast, uric acid exhibited a statistically significant negative association with VPA levels (B = −0.033, β = −0.105, *P* < 0.05), suggesting that elevated uric acid may be associated with lower blood concentrations. However, urea (B = 1.355, *P* > 0.05), retinol-binding protein (B = 0.158, *P* > 0.05) and cystatin C (B = −8.024, *P* > 0.05) were not independently associated with VPA levels.

**TABLE 5:** Results of multiple linear regression analysis of renal function variables on blood concentration.

Variable	B^a^	SE^a^	β^b^	t	*P*
Constant	54.001	9.794	NA	5.514	<0.001
Creatinine	0.452	0.103	0.201	4.391	<0.001
Uric acid	−0.033	0.015	−0.105	−2.267	0.024
Urea	1.355	0.821	0.065	1.650	0.099
Retinol-binding protein	0.158	0.158	0.045	1.000	0.317
Cystatin C	−8.024	12.230	−0.028	−0.656	0.512

Abbreviations: NA, not applicable; SE, standard error.

^a^ B denotes the unstandardized regression coefficient and SE denotes its standard error;

^b^ β denotes the standardized regression coefficient.

**FIGURE 3:**
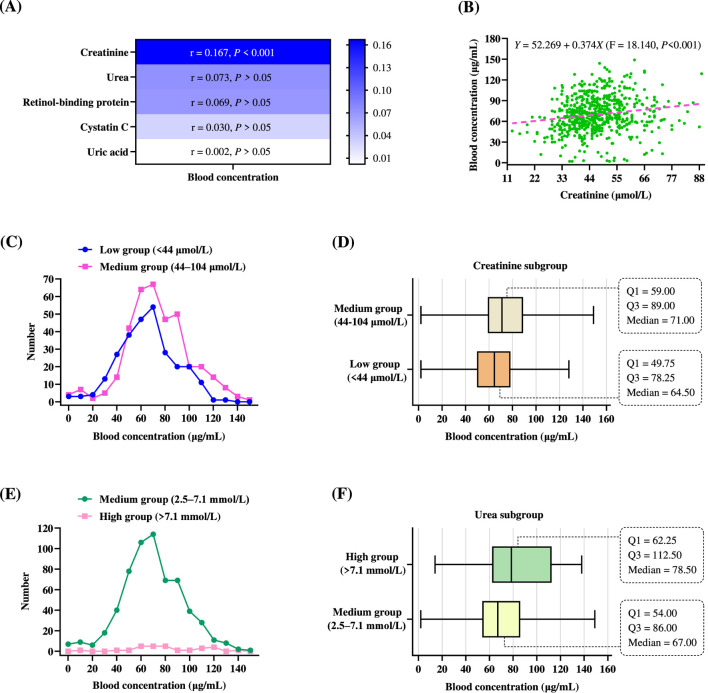
Comparison of blood concentrations across different renal function-related groups. **(A)** Heatmap of Pearson correlations between blood concentration and renal function indicators. **(B)** Scatter plot of blood concentration *versus* creatinine, with a pink dashed regression line. Each green dot represents an individual patient’s data point. **(C)** Frequency distribution of blood concentrations across different creatinine subgroups. **(D)** Box plots of blood concentrations based on creatinine grouping. Boxes represent the IQR, with vertical lines indicating the median; whiskers represent the minimum and maximum values. **(E)** Frequency distribution of blood concentrations across different urea subgroups. **(F)** Box plots of blood concentrations based on urea grouping. The box plot displays the Q1, Q3, median, and the minimum and maximum values for the two urea subgroups.

The association between blood concentration and renal function indicators—including creatinine, urea, retinol-binding protein, cystatin C, and uric acid—was initially assessed using Pearson correlation analysis (Figure 3A). Among these, creatinine demonstrated the strongest positive correlation with blood concentration (*r* = 0.167, *P* < 0.001). In contrast, urea (*r* = 0.073, *P* > 0.05), retinol-binding protein (*r* = 0.069, *P* > 0.05), cystatin C (*r* = 0.030, *P* > 0.05), and uric acid (*r* = 0.002, *P* > 0.05) showed no significant correlations with blood concentration. The univariate linear relationship between creatinine levels and blood concentration is shown in Figure 3B. A simple linear regression analysis yielded the equation: *Y* = 52.269 + 0.374*X*, where *Y* represents blood concentration and *X* denotes creatinine. The model demonstrated a statistically significant positive association between creatinine and blood concentration (F = 18.140, *P* < 0.001). Specifically, each 1 μmol/L increase in creatinine was associated with an average increase of 0.374 μg/mL in VPA levels.

#### 3.4.2 Subgroup analysis based on renal function indicators

Blood concentrations and daily dose were compared across these subgroups (Table 6). In the creatinine-based stratification, patients were categorized into a low group (<44 μmol/L) and a medium group (44–104 μmol/L). The medium group (n = 368, 57.68%) exhibited a significantly higher mean blood concentration (73.62 μg/mL; 95% CI: 70.98–76.27) compared to the low group (64.63 μg/mL; 95% CI: 61.82–67.44; *P* < 0.001). Meanwhile, the medium group required a significantly higher mean daily dose (733.90 mg/d; 95% CI: 709.02–758.78) than the low group (495.78 mg/d; 95% CI: 474.85–516.70; *P* < 0.001), suggesting that creatinine may influence both blood concentrations and dosing requirements. In the uric acid subgroups, participants were categorized into low group (<208 μmol/L), medium group (208–428 μmol/L), and high group (>428 μmol/L). The mean daily dose increased across these groups (low: 489.69 mg/d; medium: 618.33 mg/d; high: 863.39 mg/d). A significant overall difference in daily dose was observed among the three uric acid subgroups (*P* < 0.001). Post hoc pairwise comparisons with Bonferroni adjustment indicated significant differences between medium group and low group (adjusted *P* = 0.001), between high group and low group (adjusted *P* < 0.001), and between high group and medium group (adjusted *P* < 0.001). However, no significant overall difference in blood concentrations was observed among the three uric acid subgroups (*P* > 0.05). In the urea subgroups, patients in the high group (>7.1 mmol/L) had significantly higher blood concentrations (82.14 μg/mL; 95% CI: 70.81–93.47) than those in the medium group (2.5–7.1 mmol/L) (69.17 μg/mL; 95% CI: 67.18–71.15; *P* < 0.05), despite receiving similar daily dose (664.64 mg/d vs. 631.40 mg/d; *P* > 0.05). In the retinol-binding protein subgroups, compared with the medium group (25–70 mg/L), patients in the low group (<25 mg/L) were administered significantly lower daily dose (525.00 mg/d vs. 648.41 mg/d; *P* < 0.001), although blood concentrations were comparable between the two groups (69.68 μg/mL vs. 69.84 μg/mL; *P* > 0.05).

**TABLE 6:** Comparison of blood concentrations across subgroups defined by renal function indicators.

Indicator grouping	n (%)	Daily dose (mg/d)		Blood concentration (µg/mL)	
		Mean (95% CI)	*P* ^a^	Mean (95% CI)	*P* ^a^
**Creatinine subgroup**	​	​	​	​	​
Low group (<44 μmol/L)	270 (42.32)	495.78 (474.85–516.70)	<0.001	64.63 (61.82–67.44)	<0.001
Medium group (44–104 μmol/L)	368 (57.68)	733.90 (709.02–758.78)		73.62 (70.98–76.27)	
**Uric acid subgroup**	​	​	​	​	​
Low group (<208 μmol/L)	39 (6.11)	489.69 (425.07–554.31)	<0.001	67.90 (59.21–76.58)	0.318
Medium group (208–428 μmol/L)	540 (84.64)	618.33 (598.63–638.02)		69.62 (67.47–71.77)	
High group (>428 μmol/L)	59 (9.25)	863.39 (796.39–930.39)		72.88 (67.16–78.60)	
**Urea subgroup**	​	​	​	​	​
Medium group (2.5–7.1 mmol/L)	605 (94.83)	631.40 (611.48–651.32)	0.221	69.17 (67.18–71.15)	0.019
High group (>7.1 mmol/L)	28 (4.39)	664.64 (596.20–733.09)		82.14 (70.81–93.47)	
**Retinol-binding protein subgroup**	​	​	​	​	​
Low group (<25 mg/L)	79 (12.38)	525.00 (489.79–560.21)	<0.001	69.68 (63.85–75.52)	0.875
Medium group (25–70 mg/L)	559 (87.62)	648.41 (627.40–669.41)		69.84 (67.75–71.92)	

^a^ Comparisons among the three groups were conducted using the Kruskal–Wallis H test, followed by Bonferroni-corrected pairwise comparisons when appropriate. Comparisons between two groups were performed using the Mann–Whitney U test.

Abbreviation: CI, confidence interval.

The frequency distribution of blood concentrations was investigated in patient subgroups based on creatinine stratification. In the creatinine-based analysis (Figure 3C), patients in the medium group (44–104 μmol/L) exhibited a distribution centered around 50–90 μg/mL, with a peak near 70 μg/mL. The low group (<44 μmol/L) showed a distribution peak around 70 μg/mL and a broader spread toward lower blood concentrations. A larger proportion of patients in the low group had subtherapeutic VPA levels (<50 μg/mL), whereas patients in the medium group more frequently maintained blood concentrations within the therapeutic range (50–100 μg/mL). Box plots illustrating the distribution of blood concentrations in two creatinine-defined subgroups are shown in Figure 3D. In the medium group (44–104 μmol/L), the median blood concentration was 71.00 μg/mL, with the Q1 at 59.00 μg/mL and Q3 at 89.00 μg/mL. The overall distribution ranged approximately from 0 to 150 μg/mL. Conversely, the low group (<44 μmol/L) had a lower median blood concentration of 64.50 μg/mL, with Q1 and Q3 values of 49.75 μg/mL and 78.25 μg/mL, respectively. This subgroup also demonstrated a slightly narrower IQR compared to the medium group. Thus, patients with creatinine levels within the normal reference range exhibited higher median VPA blood concentrations than those with subnormal creatinine levels.

In the urea-based analysis (Figure 3E), patients in the medium group (2.5–7.1 mmol/L) demonstrated a narrow, symmetrical distribution centered around 60–70 μg/mL, with most values falling within the therapeutic range. In contrast, the high group (>7.1 mmol/L) displayed a flatter and more dispersed distribution in blood concentrations. A substantial proportion of patients in the high group had VPA levels exceeding 100 μg/mL, raising concerns about drug accumulation. The distribution of blood concentrations across urea subgroups is illustrated in Figure 3F. In the medium group (2.5–7.1 mmol/L), the median blood concentration was 67.00 μg/mL, with the Q1 at 54.00 μg/mL and Q3 at 86.00 μg/mL, yielding an IQR of 32.00 μg/mL. The total range extended from approximately 0–150 μg/mL. In comparison, the high group (>7.1 mmol/L) demonstrated a higher median blood concentration of 78.50 μg/mL, with Q1 and Q3 values of 62.25 μg/mL and 112.50 μg/mL, respectively, resulting in a substantially broader IQR of 50.25 μg/mL. This group also exhibited greater variability and a higher proportion of patients with supratherapeutic levels (>100 μg/mL). Patients with elevated urea levels displayed higher median blood concentrations and markedly greater inter-individual variability compared to those with urea levels within the normal reference range.

### 3.5 Correlation between liver function indicators and VPA blood concentration

#### 3.5.1 Multiple linear regression analysis of liver function indicators and VPA blood concentration

The relationship between liver function parameters and blood concentrations was assessed using multiple linear regression (Table 7). Alanine aminotransferase demonstrated a significant negative association with blood concentrations (B = −0.501, β = −0.236, *P* < 0.001), indicating that higher alanine aminotransferase levels were associated with lower blood concentrations. Similarly, albumin was negatively correlated with VPA levels (B = −1.278, β = −0.140, *P* < 0.001), suggesting that elevated albumin may contribute to reduced blood concentrations. In contrast, aspartate aminotransferase (B = 0.623, β = 0.239, *P* < 0.001) and globulin (B = 1.686, β = 0.253, *P* < 0.001) exhibited positive associations. Globulin had the strongest effect among all variables, implying that higher globulin levels were linked to significantly elevated blood concentrations. Alkaline phosphatase was inversely associated with VPA levels (B = −0.035, β = −0.106, *P* < 0.01), whereas glutamyl transpeptidase showed a statistically significant positive association (B = 0.306, β = 0.115, *P* < 0.01). Total bilirubin was not significantly correlated with blood concentration (*P* > 0.05). In summary, globulin, aspartate aminotransferase, and glutamyl transpeptidase were positively associated with blood concentrations, while alanine aminotransferase, albumin, and alkaline phosphatase were negatively associated.

**TABLE 7:** Results of multiple linear regression analysis of liver function variables on blood concentration.

Variable	B^a^	SE^a^	β^b^	t	*P*
Constant	75.898	17.205	NA	4.411	<0.001
Alanine aminotransferase	−0.501	0.129	−0.236	−3.874	<0.001
Aspartate aminotransferase	0.623	0.153	0.239	4.058	<0.001
Albumin	−1.278	0.370	−0.140	−3.449	<0.001
Globulin	1.686	0.274	0.253	6.157	<0.001
Total bilirubin	0.347	0.301	0.045	1.153	0.249
Alkaline phosphatase	−0.035	0.013	−0.106	−2.759	0.006
Glutamyl transpeptidase	0.306	0.111	0.115	2.763	0.006

Abbreviations: NA, not applicable; SE, standard error.

^a^ B denotes the unstandardized regression coefficient and SE denotes its standard error;

^b^ β denotes the standardized regression coefficient.

#### 3.5.2 Subgroup analysis based on liver function indicators

**FIGURE 4:**
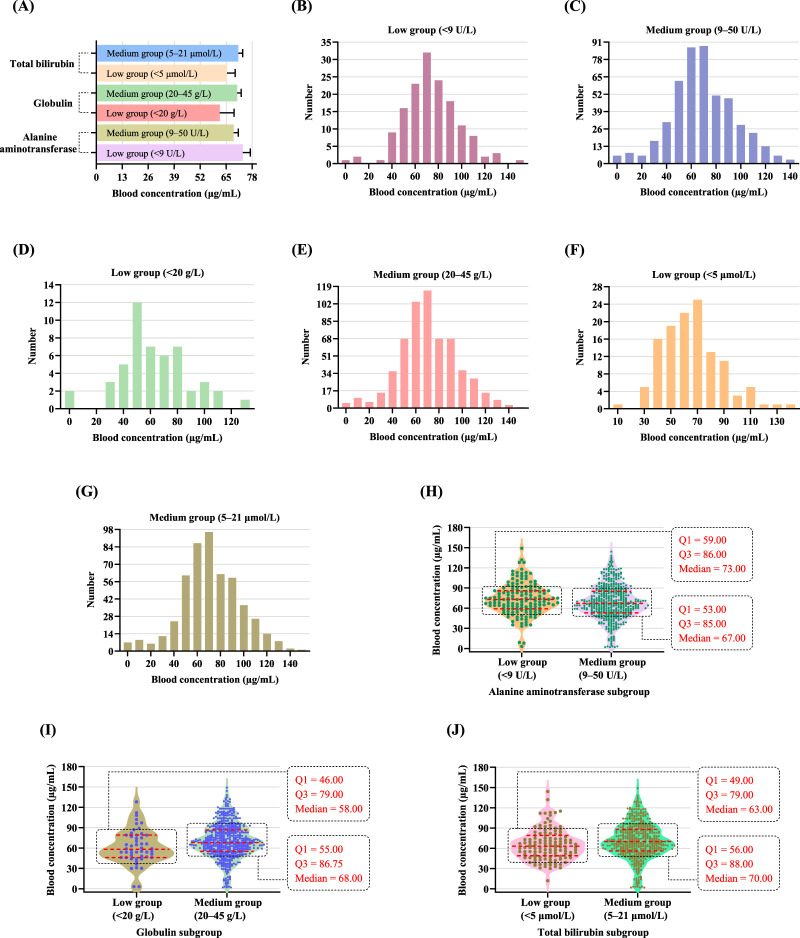
Comparison of blood concentrations among subgroups based on liver function indicators. **(A)** Mean blood concentrations across subgroups of total bilirubin, globulin, and alanine aminotransferase, with error bars indicating the 95% CI. **(B,C)** Frequency distributions of blood concentrations grouped by alanine aminotransferase levels: low group (<9 U/L) in **(B)**, and medium group (9–50 U/L) in **(C)**. **(D,E)** Frequency distributions of blood concentrations grouped by globulin levels: low group (<20 g/L) in **(D)**, and medium group (20–45 g/L) in **(E)**. **(F,G)** Frequency distributions of blood concentrations grouped by total bilirubin levels: low group (<5 μmol/L) in **(F)**, and medium group (5–21 μmol/L) in **(G)**. **(H–J)** Violin plots of blood concentrations grouped by alanine aminotransferase **(H)**, globulin **(I)**, and total bilirubin **(J)** levels. Each dot represents an individual patient’s blood concentration. The red dashed lines represent the Q1, median, and Q3, respectively.

A comparative analysis of blood concentrations and daily dose across subgroups defined by liver function indicators is presented in Table 8. For the alanine aminotransferase subgroups, patients in the low group (<9 U/L) exhibited significantly higher mean blood concentrations (73.28 μg/mL; 95% CI: 69.50–77.07) than those in the medium group (9–50 U/L) (68.76 μg/mL; 95% CI: 66.45–71.07; *P* < 0.05) (Figure 4A). However, daily dose did not differ significantly between the two groups (*P* > 0.05). For the aspartate aminotransferase subgroups, patients were categorized into low group (<15 U/L), medium group (15–40 U/L), and high group (>40 U/L). Daily dose differed significantly among the three subgroups (*P* < 0.001). Post hoc pairwise comparisons with Bonferroni correction showed that the medium group had a significantly different daily dose from the low group (adjusted *P* < 0.001), and the high group also differed significantly from the low group (adjusted *P* < 0.05), whereas no significant difference was observed between medium group and high group (adjusted *P* > 0.05). Regarding blood concentrations, a significant overall difference was also observed among the three subgroups (*P* < 0.05), with mean VPA levels of 61.78 μg/mL (95% CI: 52.28–71.27) in the low group, 69.88 μg/mL (95% CI: 67.85–71.92) in the medium group, and 80.30 μg/mL (95% CI: 70.27–90.33) in the high group. Bonferroni-adjusted *post hoc* analyses showed a significant difference between low group and high group (adjusted *P* < 0.05), while the differences between low group and medium group (adjusted *P* > 0.05) and between medium group and high group (adjusted *P* > 0.05) were not statistically significant. For the albumin subgroups, compared to the medium group (40–55 g/L), the low group (<40 g/L) received a significantly lower daily dose (503.15 mg/d vs. 638.65 mg/d; *P* < 0.01), but the difference in blood concentration was not statistically significant (73.23 μg/mL vs. 69.67 μg/mL; *P* > 0.05). In the globulin subgroups, the low group (<20 g/L) had significantly lower blood concentrations than the medium group (61.84 μg/mL vs. 70.49 μg/mL; *P* < 0.01) and also received a lower daily dose (432.88 mg/d vs. 650.15 mg/d; *P* < 0.001). For the total bilirubin subgroups, patients in the low group (<5 μmol/L) had significantly lower blood concentrations (65.42 μg/mL; 95% CI: 61.35–69.49) than those in the medium group (71.04 μg/mL; 95% CI: 68.81–73.27; *P* < 0.01), and the low group also received a significantly lower daily dose than the medium group (*P* < 0.001). For the alkaline phosphatase subgroups, although patients in the medium group (45–125 U/L) received significantly higher daily dose than those in the high group (>125 U/L) (871.55 mg/d vs. 605.62 mg/d; *P* < 0.001), no significant difference in blood concentrations was observed between the two groups (74.21 μg/mL vs. 69.31 μg/mL; *P* > 0.05).

**TABLE 8:** Comparison of blood concentrations between subgroups of different liver function indicators.

Indicator grouping	n (%)	Daily dose (mg/d)		Blood concentration (µg/mL)	
		Mean (95% CI)	*P* ^a^	Mean (95% CI)	*P* ^a^
**Alanine aminotransferase subgroup**	​	​	​	​	​
Low group (<9 U/L)	151 (23.67)	599.97 (569.73–630.22)	0.247	73.28 (69.50–77.07)	0.035
Medium group (9–50 U/L)	479 (75.08)	640.58 (617.15–664.01)		68.76 (66.45–71.07)	
**Aspartate aminotransferase subgroup**	​	​	​	​	​
Low group (<15 U/L)	40 (6.27)	817.50 (734.67–900.33)	<0.001	61.78 (52.28–71.27)	0.019
Medium group (15–40 U/L)	571 (89.50)	617.90 (598.62–637.19)		69.88 (67.85–71.92)	
High group (>40 U/L)	27 (4.23)	681.93 (545.12–818.73)		80.30 (70.27–90.33)	
**Albumin subgroup**	​	​	​	​	​
Low group (<40 g/L)	26 (4.08)	503.15 (457.45–548.86)	0.008	73.23 (64.80–81.66)	0.523
Medium group (40–55 g/L)	612 (95.92)	638.65 (618.88–658.41)		69.67 (67.66–71.69)	
**Globulin subgroup**	​	​	​	​	​
Low group (<20 g/L)	50 (7.84)	432.88 (382.16–483.60)	<0.001	61.84 (54.73–68.95)	0.009
Medium group (20–45 g/L)	588 (92.16)	650.15 (630.40–669.91)		70.49 (68.46–72.53)	
**Total bilirubin subgroup**	​	​	​	​	​
Low group (<5 μmol/L)	123 (19.28)	532.85 (495.10–570.60)	<0.001	65.42 (61.35–69.49)	0.004
Medium group (5–21 μmol/L)	511 (80.09)	655.86 (634.25–677.47)		71.04 (68.81–73.27)	
**Alkaline phosphatase subgroup**	​	​	​	​	​
Medium group (45–125 U/L)	66 (10.34)	871.55 (799.73–943.36)	<0.001	74.21 (67.47–80.95)	0.205
High group (>125 U/L)	572 (89.66)	605.62 (587.13–624.10)		69.31 (67.26–71.36)	

^a^ Comparisons among the three groups were conducted using the Kruskal–Wallis H test, followed by Bonferroni-corrected pairwise comparisons when appropriate. Comparisons between two groups were performed using the Mann–Whitney U test.

Abbreviation: CI, confidence interval.

The frequency distribution of blood concentrations in patients with alanine aminotransferase levels below 9 U/L is illustrated in Figure 4B. The most frequently observed blood concentration was approximately 70 μg/mL, with a peak count of 30–35 patients. Most values clustered within the therapeutic range of 50–100 μg/mL. Only a small proportion of patients had subtherapeutic (<50 μg/mL) or supratherapeutic (>100 μg/mL) blood concentrations, indicating that the majority of patients achieved optimal blood concentrations. Figure 4C shows the frequency distribution of blood concentrations in patients with alanine aminotransferase levels within the reference range (9–50 U/L). The distribution appeared symmetric, centered within the therapeutic range. Most blood concentrations fell between 50 and 100 μg/mL, with a prominent peak between 60 and 70 μg/mL. The 60–70 μg/mL interval accounted for the largest patient group, with two adjacent bars each approaching 90 individuals. Notably, a considerable proportion of patients had blood concentrations either above 100 μg/mL or below 50 μg/mL. Violin plots of blood concentrations grouped by alanine aminotransferase levels are shown in Figure 4H. In the low group (<9 U/L), the median blood concentration was 73.00 μg/mL, with the IQR was 27.00 μg/mL. In comparison, the medium group (9–50 U/L) exhibited a slightly lower median blood concentration of 67.00 μg/mL, with Q1 = 53.00 μg/mL and Q3 = 85.00 μg/mL, yielding a wider IQR of 32.00 μg/mL. Although both subgroups showed median values within the therapeutic range of 50–100 μg/mL, the low group (<9 U/L) demonstrated slightly higher central tendency and a more concentrated distribution. Thus, patients with alanine aminotransferase levels below the reference range tended to exhibit slightly higher and more centralized blood concentrations compared to those with normal alanine aminotransferase levels.

The frequency distribution of blood concentrations among patients with low globulin levels (<20 g/L) is shown in Figure 4D. The distribution of blood concentrations ranged from approximately 0–130 μg/mL, with a peak around 50 μg/mL. A substantial number of individuals had blood concentrations below 50 μg/mL, and only a small proportion exceeded 100 μg/mL. Overall, patients in the low globulin group (<20 g/L) exhibited relatively low and dispersed blood concentrations, with a prominent tendency toward subtherapeutic levels. Figure 4E depicts the frequency distribution of blood concentrations in patients with globulin levels within the reference range (20–45 g/L). The distribution demonstrated a unimodal, approximately normal pattern centered within the therapeutic window, with the highest frequency observed in the 60–70 μg/mL interval. The majority of values fell between 50 and 100 μg/mL, consistent with the recommended therapeutic range. A few patients had blood concentrations below 50 μg/mL or above 100 μg/mL. So, patients in the medium group (20–45 g/L) exhibited a well-centered distribution of blood concentrations within the therapeutic range, with minimal deviation toward sub- or supratherapeutic levels. The box plots for the low group (<20 g/L) and the medium group (20–45 g/L) are shown in Figure 4I. In the low group (<20 g/L), the median blood concentration was 58.00 μg/mL, with the Q1 at 46.00 μg/mL and Q3 at 79.00 μg/mL, yielding an IQR of 33.00 μg/mL. In comparison, the medium group (20–45 g/L) exhibited a higher median blood concentration of 68.00 μg/mL, with Q1 and Q3 values of 55.00 μg/mL and 86.75 μg/mL, respectively, resulting in an IQR of 31.75 μg/mL. The distribution density and individual data points showed that a larger proportion of patients in the medium group maintained blood concentrations within the recommended therapeutic range (50–100 μg/mL). In summary, patients with globulin levels within the normal reference range (20–45 g/L) had markedly higher median blood concentrations than those with lower globulin levels, with a slightly more concentrated distribution of blood concentrations.

The frequency distribution of blood concentrations in patients with low total bilirubin levels (<5 μmol/L) is illustrated in Figure 4F. The blood concentrations ranged from approximately 10–140 μg/mL, with the peak of the distribution occurring at approximately 70 μg/mL. Most patients had blood concentrations between 50 and 100 μg/mL, consistent with the recommended therapeutic range. Subtherapeutic blood concentrations (<50 μg/mL) were observed in a considerable proportion of patients, primarily clustering between 30 and 40 μg/mL, whereas only a few patients exhibited concentrations >100 μg/mL. The frequency distribution of blood concentrations in patients with total bilirubin levels within the normal reference range (5–21 μmol/L) is shown in Figure 4G. The distribution exhibited a unimodal, approximately normal pattern, with blood concentrations ranging from 0 to 150 μg/mL and the highest frequency observed around 70 μg/mL. A majority of values fell within the recommended therapeutic range (50–100 μg/mL), particularly clustered between 50 and 90 μg/mL. Only a small proportion of patients had subtherapeutic concentrations (<50 μg/mL) or supratherapeutic concentrations (>100 μg/mL). Figure 4J presents violin plots of blood concentrations stratified by total bilirubin levels. In the low group (<5 μmol/L), the median blood concentration was 63.00 μg/mL, with a Q1 of 49.00 μg/mL and a Q3 of 79.00 μg/mL, yielding an IQR of 30.00 μg/mL. Although the distribution was relatively centered, it was slightly skewed toward the lower end of the therapeutic range, and a considerable number of patients exhibited subtherapeutic levels (<50 μg/mL). In contrast, the medium group (5–21 μmol/L) showed a higher median blood concentration of 70.00 μg/mL, with Q1 and Q3 values of 56.00 μg/mL and 88.00 μg/mL, respectively, resulting in an IQR of 32.00 μg/mL. This group exhibited a distribution shifted upward and more tightly clustered within the therapeutic window, with some patients displaying values outside the target range.

### 3.6 Effect of combination therapy on VPA blood concentration

Patients were categorized into three groups based on the type of co-administered antiepileptic drugs (Table 9). Combination therapy was defined as the concurrent use of VPA with one, and only one, additional antiepileptic drug. Regimens involving VPA in combination with two or more additional antiepileptic drugs, as well as those including concomitant non-antiepileptic medications, were excluded from the analysis. The single drug group received VPA (n = 275) as monotherapy. The enzyme inducer group comprised patients treated with VPA in combination with known enzyme-inducing agents, specifically oxcarbazepine (n = 26). The non-enzyme inducer/non-enzyme inhibitor group included those receiving VPA alongside agents with limited effects on hepatic enzyme induction or inhibition, namely, levetiracetam (n = 124), lamotrigine (n = 60), lacosamide (n = 65), perampanel (n = 74), zonisamide (n = 2), or topiramate (n = 12). The mean daily dose of VPA was comparable among the three groups, with values of 626.20 mg/d (95% CI: 595.84–656.56) in the single drug group, 651.85 mg/d (95% CI: 553.59–750.10) in the enzyme inducer group, and 637.34 mg/d (95% CI: 611.71–662.97) in the non-enzyme inducer/non-enzyme inhibitor group, showing no statistically significant difference (*P* > 0.05). Similarly, blood concentration levels did not differ significantly across medication subgroups. The mean blood concentrations were 69.35 μg/mL (95% CI: 66.32–72.37), 65.65 μg/mL (95% CI: 55.77–75.54), and 70.52 μg/mL (95% CI: 67.83–73.21) in the single drug, enzyme inducer, and non-enzyme inducer/non-enzyme inhibitor groups, respectively, with comparable median values and IQRs. No significant overall difference in blood concentration was observed among the three groups (*P* > 0.05).

**TABLE 9:** Comparison of daily dose and blood concentration based on combination therapy situation.

Medication grouping	n (%)	Daily dose (mg/d)	Blood concentration (µg/mL)				
		Mean (95% CI)	Mean (95% CI)	Median	Q1	Q3	IQR
**Drug subgroups**	​	​	​	​	​	​	​
Single drug group	275 (43.10)	626.20 (595.84–656.56)	69.35 (66.32–72.37)	67.00	53.00	86.00	33.00
Enzyme inducer group	26 (4.08)	651.85 (553.59–750.10)	65.65 (55.77–75.54)	64.00	48.25	82.50	34.25
Non-enzyme inducer/non-enzyme inhibitor group	337 (52.82)	637.34 (611.71–662.97)	70.52 (67.83–73.21)	69.00	56.00	86.00	30.00
**Kruskal–Wallis H test**	​	​	​				
H	NA	1.395	1.169				
*P*	NA	0.498	0.557				

Abbreviations: NA, not applicable; CI, confidence interval; Q1, first quartile; Q3, third quartile; IQR, interquartile range.

### 3.7 Correlation between electrolyte indicators and VPA blood concentration

#### 3.7.1 Linear regression analysis of electrolyte indicators and VPA blood concentration

The results of a multiple linear regression analysis assessing the relationship between electrolyte levels and VPA blood concentrations are presented in Table 10. Among the five electrolytes examined, calcium level was inversely associated with blood concentration (B = −40.457, β = −0.130, *P* < 0.01). Similarly, phosphorus level also showed a significant negative association (B = −18.814, β = −0.135, *P* < 0.001), suggesting that elevated phosphorus levels independently predicted lower VPA blood concentrations. In contrast, potassium (B = 2.915, *P* > 0.05), sodium (B = 0.318, *P* > 0.05), and chloride (B = −0.504, *P* > 0.05) levels were not significantly associated with blood concentrations. In summary, higher calcium and phosphorus levels were independently associated with reduced blood concentrations, while other electrolytes showed no significant effect.

**TABLE 10:** Results of multiple linear regression analysis of electrolyte variables on blood concentration.

Variable	B^a^	SE^a^	β^b^	t	*P*
Constant	194.238	71.179	NA	2.729	0.007
Potassium	2.915	3.173	0.038	0.919	0.359
Sodium	0.318	0.434	0.031	0.734	0.463
Chloride	−0.504	0.495	−0.044	−1.017	0.309
Calcium	−40.457	12.910	−0.130	−3.134	0.002
Phosphorus	−18.814	5.545	−0.135	−3.393	<0.001

Abbreviations: NA, not applicable; SE, standard error.

^a^ B denotes the unstandardized regression coefficient and SE denotes its standard error;

^b^ β denotes the standardized regression coefficient.

**FIGURE 5:**
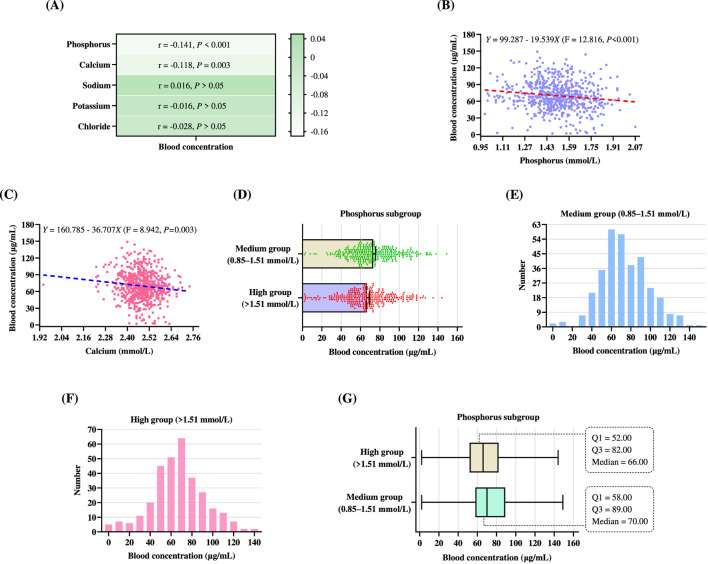
Comparison of blood concentrations among different electrolyte groups. **(A)** Heatmap of Pearson correlations between blood concentration and electrolyte indicators. **(B)** Scatter plot of the relationship between blood concentration and phosphorus level, with a fitted linear regression line (red dashed line). Each light blue dot represents an individual patient’s data point. **(C)** Scatter plot of the relationship between blood concentration and calcium level, with blue dashed line representing regression trend line and pink dots representing individual data points. **(D)** Mean blood concentrations in the two phosphorus subgroups, with error bars representing the 95% CI. **(E,F)** Frequency distributions of blood concentrations grouped by phosphorus levels: medium group (0.85–1.51 mmol/L) in **(E)**, and high group (>1.51 mmol/L) in **(F)**. **(G)** Box plot of blood concentrations distribution based on phosphorus level grouping. The box plot shows the Q1, Q3, median, and the minimum and maximum values for the two phosphorus subgroups.

We further performed Pearson correlation analyses between blood concentrations and five electrolyte parameters, as shown in Figure 5A. Among these variables, phosphorus exhibited a statistically significant negative correlation with blood concentrations (*r* = −0.141, *P* < 0.001), indicating that higher phosphorus levels were associated with lower VPA blood concentrations. Similarly, calcium also showed a significant negative correlation with blood concentrations (*r* = −0.118, *P* < 0.01). In contrast, no statistically significant correlations were observed for sodium (*r* = 0.016, *P* > 0.05), potassium (*r* = −0.016, *P* > 0.05), or chloride (*r* = −0.028, *P* > 0.05), suggesting limited or no linear association between these electrolytes and VPA blood concentrations. The findings from these Pearson correlation analyses were entirely consistent with those of the multiple linear regression analysis.

Based on the results of the Pearson correlation analysis, we further investigated the univariate linear relationship between phosphorus levels and blood concentrations (Figure 5B). Linear regression analysis revealed a statistically significant inverse association between the two variables, described by the equation: *Y* = 99.287–19.539*X* (F = 12.816, *P* < 0.001), where *Y* represents the blood concentration and *X* denotes the phosphorus level. The negative slope indicates that for each 1 mmol/L increase in phosphorus level, blood concentration decreases by approximately 19.539 μg/mL. Although the individual data points show substantial variability, the fitted red dashed regression line demonstrates a clear downward trend. Figure 5C illustrates a scatter plot demonstrating the relationship between calcium levels and blood concentrations. Linear regression analysis revealed a statistically significant inverse association between the two variables, expressed by the regression equation: *Y* = 160.785–36.707*X* (F = 8.942, *P* < 0.01), where *Y* denotes the blood concentration and X represents the calcium level. The negative slope indicates that for each 1 mmol/L increase in calcium level, the predicted blood concentration decreased by approximately 36.707 μg/mL.

#### 3.7.2 Subgroup analysis based on electrolyte levels

A subgroup analysis was performed to assess differences in the daily dose and blood concentrations according to electrolyte levels (Table 11). For phosphorus, patients in the high group (>1.51 mmol/L) exhibited significantly lower mean blood concentrations compared to those in the medium group (0.85–1.51 mmol/L) (66.50 μg/mL vs. 73.01 μg/mL; *P* < 0.01) (Figure 5D). Meanwhile, the high group (>1.51 mmol/L) received significantly lower daily dose (561.46 mg/d vs. 702.15 mg/d; *P* < 0.001). Regarding calcium, although patients in the high group (>2.52 mmol/L) had slightly lower daily dose than those in the medium group (2.11–2.52 mmol/L) (603.01 mg/d vs. 644.07 mg/d), the difference was not statistically significant (*P* > 0.05). Similarly, no significant difference in blood concentration was observed between the two groups (66.83 μg/mL vs. 70.90 μg/mL; *P* > 0.05). For sodium, the low group (<137 mmol/L) and the medium group (137–147 mmol/L) did not differ significantly in either daily dose or blood concentration (both *P* > 0.05). For chlorine, although daily dose differed significantly between the medium group (99–110 mmol/L) and the high group (>110 mmol/L) (*P* < 0.05), blood concentrations did not differ significantly between the two groups (*P* > 0.05).

**TABLE 11:** Comparison of blood concentrations between subgroups of different electrolyte levels.

Indicator grouping	n (%)	Daily dose (mg/d)		Blood concentration (µg/mL)	
		Mean (95% CI)	*P* ^a^	Mean (95% CI)	*P* ^a^
**Sodium subgroup**	​	​	​	​	​
Low group (<137 mmol/L)	35 (5.49)	555.51 (483.91–627.12)	0.076	69.03 (60.61–77.44)	0.736
Medium group (137–147 mmol/L)	592 (92.79)	638.16 (618.24–658.08)		69.95 (67.90–72.00)	
**Chlorine subgroup**	​	​	​	​	​
Medium group (99–110 mmol/L)	613 (96.08)	637.38 (617.79–656.98)	0.013	69.78 (67.77–71.80)	0.709
High group (>110 mmol/L)	22 (3.45)	509.91 (430.12–589.69)		70.95 (60.88–81.03)	
**Calcium subgroup**	​	​	​	​	​
Medium group (2.11–2.52 mmol/L)	467 (73.20)	644.07 (621.94–666.21)	0.081	70.90 (68.57–73.23)	0.198
High group (>2.52 mmol/L)	170 (26.65)	603.01 (564.70–641.33)		66.83 (63.21–70.45)	
**Phosphorus subgroup**	​	​	​	​	​
Medium group (0.85–1.51 mmol/L)	325 (50.94)	702.15 (674.18–730.12)	<0.001	73.01 (70.33–75.68)	0.003
High group (>1.51 mmol/L)	313 (49.06)	561.46 (537.73–585.19)		66.50 (63.66–69.35)	

Abbreviation: CI, confidence interval.

^a^ Comparisons between two groups were performed using the Mann–Whitney U test.

The frequency distribution of blood concentrations in patients with phosphorus levels within the reference range (0.85–1.51 mmol/L) is illustrated in Figure 5E. Two peak frequencies were observed at the 60 μg/mL and 70 μg/mL intervals, with two additional peaks occurring at 80 μg/mL and 90 μg/mL, indicating that most blood concentrations in this group were within the therapeutic range of 50–100 μg/mL. However, a considerable number of patients still exhibited subtherapeutic (<50 μg/mL) or supratherapeutic (>100 μg/mL) concentrations. Figure 5F presents the frequency distribution of blood concentrations in patients with phosphorus levels above the reference range (>1.51 mmol/L). The most commonly observed blood concentration was 70 μg/mL, encompassing approximately 65 patients. Additional clustering occurred in the 50–60 μg/mL and 80–90 μg/mL ranges. Notably, some patients in this subgroup exhibited subtherapeutic concentrations (<50 μg/mL) or supratherapeutic levels (>100 μg/mL). Figure 5G presents a box plot of blood concentrations stratified by phosphorus levels, comparing the medium group (0.85–1.51 mmol/L) and the high group (>1.51 mmol/L). The median blood concentration in the medium group (0.85–1.51 mmol/L) was 70.00 μg/mL, with an IQR of 31.00 μg/mL (Q1 = 58.00 μg/mL, Q3 = 89.00 μg/mL). In comparison, the high group (>1.51 mmol/L) had a lower median blood concentration of 66.00 μg/mL and a narrower IQR of 30.00 μg/mL (Q1 = 52.00 μg/mL, Q3 = 82.00 μg/mL). Although blood concentrations in both groups primarily fell within the therapeutic range (50–100 μg/mL), patients with elevated phosphorus levels tended to exhibit slightly lower blood concentrations. These findings further support the inverse association between phosphorus levels and VPA blood concentrations.

## 4 Discussion

This study demonstrates that epilepsy management among pediatric patients with epilepsy is primarily conducted in outpatient settings, particularly through pediatric clinics. This pattern reflects the chronic yet controllable nature of epilepsy in this population, where most stable patients require only routine monitoring and medication adjustments. The extremely low rate of emergency visits further supports the notion of generally well-controlled disease status. In this cohort of 638 pediatric patients with epilepsy, the predominance of male patients and the age distribution skewed toward the 6–12-year group are consistent with established epidemiological patterns of childhood epilepsy (Specchio et al., 2022).

The mean VPA blood concentration was 69.82 μg/mL, falling well within the therapeutic window (50–100 μg/mL), which indicates effective dose adjustment under routine clinical monitoring. The mean daily dose of VPA observed in our cohort was 633.13 mg/d, reflecting dosing practices in pediatric patients with epilepsy. Liver and renal function indicators were largely within the normal ranges, suggesting good tolerability of VPA in this pediatric population. Notably, the only abnormal biochemical parameter was alkaline phosphatase, which exhibited a mean value significantly exceeding the upper normal limit; thus, its elevation warrants cautious interpretation in the context of long-term therapy. Electrolyte levels—including sodium, potassium, calcium, and chlorine—remained generally stable, indicating minimal impact of VPA on electrolyte homeostasis. The mean phosphorus level was close to the upper limit of the normal reference range, which may reflect age-related metabolic variations or dietary influences. Finally, more than half of the patients received combination therapy, among whom only a small proportion were treated with enzyme-inducing agents. This pattern suggests a clinical preference for regimens with limited involvement of enzyme-inducing or enzyme-inhibiting agents, aiming to reduce pharmacokinetic interactions and maintain stable VPA blood concentrations. Overall, the results support the effectiveness and tolerability of VPA as either monotherapy or part of a combination regimen in pediatric epilepsy management, with biochemical monitoring providing reassurance regarding organ function and systemic safety (Davis et al., 1994; Rosati et al., 2015).

### 4.1 Influence of daily dose on VPA blood concentration

The distribution analysis of VPA blood concentrations and daily dose offers valuable insight into the clinical management of pediatric epilepsy. Most patients achieved blood concentrations within the recommended therapeutic range (50–100 μg/mL), with a mode centered around 70 μg/mL. This indicates effective dose individualization and adherence to TDM practices in the studied cohort (Patsalos et al., 2008). The distribution of daily dose clustered primarily between 450 and 900 mg/d, with approximately 500 and 800 mg/d being the most commonly prescribed doses. This pattern corresponds with empirical dosing strategies based on patient age and clinical response. The observed positive correlation between daily dose and VPA blood concentration (*r* = 0.319, *P* < 0.001) indicates that daily dose is an important determinant of blood levels in pediatric patients with epilepsy (Hsu et al., 2024). However, the moderate strength of this correlation indicates that daily dose alone does not fully explain the interindividual variability in blood concentration. Furthermore, the wide vertical dispersion of data points at fixed dose levels, particularly around 500, 750, and 1,000 mg/d, underscores the limitations of fixed-dose strategies in pediatric patients with epilepsy (Xu et al., 2018). This inter-individual variability in blood concentrations among patients receiving similar daily doses further highlights the importance of individualized TDM to optimize treatment efficacy and safety.

Among subgroups stratified by daily dose, both the mean and median blood concentrations increased progressively from the low group (<500 mg/d) to the high group (>800 mg/d), with statistically significant differences observed across all pairwise comparisons. These findings support the clinical expectation that higher daily doses are associated with higher blood concentrations, as reflected by increased mean and median values. Notably, the high group (>800 mg/d) exhibited the widest IQR and the flattest density distribution, indicating substantial interindividual pharmacokinetic variability. This variability may be attributed to age-related differences in hepatic metabolism, plasma protein binding, and co-medication effects—all of which are particularly relevant in pediatric patients with epilepsy undergoing continuous physiological development (Methaneethorn, 2018; Radovanović et al., 2024; Wang et al., 2024). In conclusion, VPA blood concentrations were generally correlated with daily dose in this study, although substantial interindividual variability remained.

### 4.2 Influence of age and gender on VPA blood concentration

The results demonstrate a monotonic increase in daily dose across the three age groups, supporting the notion that clinicians adjust the daily dose upward with advancing age in clinical practice. Notably, the early childhood group (<6 years), which received the lowest mean daily dose, also exhibited the lowest mean and median blood concentrations. First, age-related differences in VPA blood concentrations may be partly attributable to developmental changes in the enzymes involved in VPA metabolism. VPA is eliminated primarily through glucuronidation, mitochondrial β-oxidation, and, to a lesser extent, CYP-mediated oxidation. Glucuronidation is the principal elimination pathway and is mediated by UGT enzymes, which undergo developmental maturation (Strassburg et al., 2002). In infants, UGT expression and glucuronidation capacity remain immature and are generally lower than those in older children, adolescents, and adults (Reith et al., 2000; Guerrini, 2006; Monostory et al., 2019). Consequently, children younger than 2 years of age have less mature hepatic drug-metabolizing enzyme systems, which may increase their susceptibility to VPA-associated hepatotoxicity (Ghodke-Puranik et al., 2013). Second, children aged 2–6 years often exhibit higher drug clearance than adults (Guerrini, 2006). Third, VPA is highly protein-bound, and age-related differences in plasma protein composition may affect its free fraction and distribution. Together, these factors provide plausible explanations for the distinctly lower blood concentrations observed in children younger than 6 years. Therefore, a cautious dosing strategy that includes lower initial daily doses and close TDM is recommended for children in the early childhood group (<6 years). Moreover, the distributions of blood concentrations in the middle childhood group (6–12 years) and adolescent group (>12 years) were near-normal and centered within the therapeutic range, indicating greater pharmacokinetic stability at the population level after 6 years of age. The similarity in central tendency and IQR between these two groups may reflect a plateau in VPA metabolic capacity beyond 6 years of age, which could serve as a reference point for individualized dosing strategies. Importantly, although most patients in the middle childhood group (6–12 years) and adolescent group (>12 years) achieved therapeutic concentrations (50–100 μg/mL), a small subset still exhibited supratherapeutic levels (>100 μg/mL), underscoring the continued need for TDM even in age groups with relatively more predictable pharmacokinetics. Conversely, the early childhood group (<6 years) had a higher proportion of subtherapeutic concentrations (<50 μg/mL), further emphasizing the necessity of individualized titration in younger children. Across age groups, the IQR of VPA blood concentrations increased progressively with age, indicating greater interindividual variability in the middle childhood group (6–12 years) and adolescent group (>12 years). This finding suggests that although pharmacokinetics may appear more stable at the population level in these age groups, substantial individual variability persists. Such variability may be due to factors including genetic polymorphisms, concomitant medications, and dietary influences.

These age-related patterns are broadly consistent with findings from previous real-world studies. In a recent large real-world study of psychiatric inpatients, Avrahami et al. identified age as an independent predictor of VPA levels (Avrahami et al., 2024). Notably, adolescent psychiatric patients exhibited higher dose-adjusted VPA levels than adults. Although that cohort did not include children younger than 12 years of age, the findings similarly underscore the importance of age-dependent variability in VPA pharmacokinetics. Taken together, these results suggest that developmental stage, rather than clinical indication alone, plays a critical role in determining VPA levels. Therefore, individualized dosing strategies and careful TDM are essential in both neurological and psychiatric pediatric populations.

An important observation was that the mean daily dose was higher in males than in females, whereas VPA blood concentrations were generally comparable between the two groups. This suggests that, in routine practice, after dose individualization guided by clinical response, adverse effects, and TDM, we did not observe a meaningful gender-related difference in VPA blood concentrations in this pediatric cohort. Notably, the IQR of VPA blood concentrations was wider in females than in males, indicating greater variability among female patients. Potential explanations include greater inter-individual heterogeneity related to differences in body composition, hormone fluctuations, protein-binding status, or metabolic capacity.

### 4.3 Influence of renal function indicators on VPA blood concentration

Of all renal function indicators analyzed, creatinine showed the strongest and most consistent association with VPA blood concentrations. Both univariate and multivariate linear regression analyses revealed that elevated creatinine levels were significantly associated with higher VPA blood concentrations, consistent with previous research findings (Hsu et al., 2024). Furthermore, stratified analysis indicated that patients within the normal creatinine reference range exhibited higher median and mean VPA blood concentrations than those with subnormal creatinine levels, while also receiving higher daily doses. Frequency distribution and box plot analyses further confirmed that the medium group (44–104 μmol/L) was more likely to maintain VPA blood concentrations within the therapeutic range. Uric acid exhibited a negative association with VPA blood concentrations, although the effect size was modest. The underlying mechanism remains unclear but may involve metabolic or transporter-related interactions that warrant further investigation. In contrast, urea, retinol-binding protein, and cystatin C were not significantly independently associated with VPA levels. These findings suggest that, within this cohort, these biomarkers may not reliably reflect the pharmacokinetic behavior of VPA and may be influenced by additional confounding factors. In summary, these results suggest that renal function, particularly glomerular filtration capacity as indirectly reflected by creatinine levels, may play a clinically relevant role in the pharmacokinetics of VPA. Collectively, these findings support the utility of routine renal function monitoring, with particular attention to creatinine levels, in pediatric patients with epilepsy receiving VPA, in order to inform individualized dosing and help maintain blood concentrations within the therapeutic range (Anguissola et al., 2023).

### 4.4 Influence of liver function indicators on VPA blood concentration

Alanine aminotransferase demonstrated a significant inverse correlation with VPA blood concentrations. Patients with alanine aminotransferase levels below 9 U/L exhibited higher mean blood concentrations than those within the normal reference range (9–50 U/L). Violin plots and frequency distribution analyses illustrated that patients with low alanine aminotransferase levels (<9 U/L) tended to have fewer blood concentration values outside the therapeutic range. In contrast, aspartate aminotransferase was positively correlated with VPA blood concentrations. In the analysis of blood concentrations stratified by aspartate aminotransferase levels, although the overall difference among the groups was statistically significant, Bonferroni-adjusted *post hoc* analyses showed that neither the low group (<15 U/L) nor the high group (>40 U/L) differed significantly from the medium group (15–40 U/L). Alkaline phosphatase was negatively associated with VPA levels, whereas glutamyl transpeptidase was positively associated with VPA levels, although the underlying mechanisms remain unclear.

Globulin emerged as the strongest positive predictor of VPA blood concentrations, as consistently demonstrated across both regression analyses and violin plot comparisons. Patients with globulin levels within the normal range (20–45 g/L) demonstrated higher median VPA blood concentrations and a more centralized distribution within the therapeutic range, accompanied by higher daily doses, compared with those with low globulin levels (<20 g/L). Albumin was negatively associated with VPA blood concentrations in the multiple linear regression analysis. Because VPA is highly protein bound and primarily binds to albumin in plasma, variation in albumin levels may be associated with differences in VPA disposition and measured steady-state total trough concentrations. However, when patients were stratified by albumin level, daily dose differed significantly across subgroups, whereas VPA blood concentrations did not.

Total bilirubin was not significantly correlated with VPA blood concentrations in the regression model; however, subgroup analysis revealed that patients within the reference range (5–21 μmol/L) exhibited significantly higher blood concentrations and a more symmetrical distribution within the therapeutic window compared to those with total bilirubin levels below 5 μmol/L. These findings suggest that lower total bilirubin levels may reflect underlying physiological or metabolic differences associated with VPA blood concentrations, although the exact mechanism remains unclear.

This study systematically examined the relationship between liver function parameters and VPA blood concentrations, emphasizing the impact of specific biochemical markers on VPA blood concentrations. The results indicate that several liver function indicators, particularly alanine aminotransferase and globulin, are significantly associated with inter-individual differences in VPA blood concentrations, with important implications for TDM and individualized dosing strategies. Collectively, these findings highlight the critical role of liver function evaluation in interpreting VPA pharmacokinetics. While markers such as alanine aminotransferase and globulin showed relatively consistent associations with VPA blood concentrations, other indicators exhibited weaker or more variable relationships. Incorporating liver function status into the clinical interpretation of VPA blood concentrations may enhance dose individualization and optimize therapeutic monitoring, particularly in pediatric or hepatically impaired populations (Meseguer et al., 2021).

### 4.5 Influence of combination therapy on VPA blood concentration

Daily dose and blood concentration in the enzyme inducer group did not differ significantly from those observed in the single drug group. Because the enzyme inducer group consisted exclusively of patients receiving VPA in combination with oxcarbazepine, the findings for this subgroup should be interpreted as reflecting this specific combination rather than enzyme-inducing agents as a whole. Therefore, the following discussion focuses mainly on oxcarbazepine. Oxcarbazepine is a prodrug that is rapidly converted *in vivo* to its pharmacologically active metabolite, the 10-monohydroxy derivative (MHD). Both oxcarbazepine and MHD can inhibit CYP2C19 and induce CYP3A4/5 (Karaźniewicz-Łada et al., 2021). However, because MHD is only a weak inducer of UGT enzymes, oxcarbazepine is generally considered to have minimal effects on drugs that are primarily eliminated by UGT-mediated glucuronidation, such as VPA. In addition, this finding may also be partly attributable to the relatively small sample size of the enzyme inducer group, which may have limited the statistical power to detect subtle differences. Although no significant differences in blood concentrations were observed between the single drug group and the enzyme inducer group, clinicians should remain vigilant for potential pharmacokinetic interactions that may necessitate dose adjustments. Future studies with larger sample sizes are warranted to further clarify these interaction patterns and refine clinical dosing recommendations. Similarly, patients in the non-enzyme inducer/non-enzyme inhibitor group—who received VPA alongside agents such as levetiracetam, lamotrigine, lacosamide, perampanel, zonisamide, or topiramate—exhibited daily doses and blood concentrations comparable to those in the single drug group. These agents have a relatively limited impact on hepatic enzyme activity and thus exert only minor effects on VPA pharmacokinetics. As a result, both the administered doses and achieved concentrations remained stable and within the expected therapeutic range.

### 4.6 Influence of electrolyte indicators on VPA blood concentration

The inverse relationship between phosphorus levels and VPA blood concentrations may be partially attributed to the physiological interplay between phosphate metabolism and hepatic or renal drug elimination pathways. Because VPA is metabolized mainly in the liver and eliminated predominantly as metabolites in urine, alterations in renal or metabolic status may potentially influence its disposition. Subgroup analyses further clarified the clinical relevance of this association. Patients with elevated phosphorus levels (>1.51 mmol/L) received lower daily doses and exhibited lower blood concentrations, suggesting altered drug distribution or clearance mechanisms that warrant further investigation. Calcium levels were significantly negatively associated with VPA blood concentrations, which may reflect similar underlying regulatory mechanisms. Calcium plays a role in drug-binding processes and intracellular signaling pathways that influence hepatic metabolism. VPA is highly protein-bound, and fluctuations in serum calcium levels may be associated with changes in protein-binding status, thereby potentially influencing VPA disposition. In contrast, no significant associations were observed between VPA blood concentrations and levels of sodium, potassium, or chloride. This suggests that these electrolytes may not substantially affect VPA pharmacokinetics under physiological conditions, or that compensatory mechanisms mitigate their potential impact.

Taken together, this study comprehensively investigated the associations between electrolyte parameters and VPA blood concentrations in pediatric patients with epilepsy. Notably, elevated levels of phosphorus and calcium were independently associated with significantly lower VPA blood concentrations, as demonstrated by both multiple linear regression and Pearson correlation analyses. The consistency of these results across statistical methods strengthens the validity of the observed associations. These findings underscore the importance of evaluating electrolyte status—particularly phosphorus and calcium levels—when optimizing VPA therapy. Routine monitoring of these parameters in pediatric patients with epilepsy receiving VPA may enhance individualized dosing strategies and reduce the risk of subtherapeutic blood concentrations or adverse effects. Further prospective studies are needed to confirm these associations in larger, more heterogeneous pediatric populations and to explore the underlying biological mechanisms.

### 4.7 Strengths and limitations

The present study is strengthened by its comprehensive evaluation of the associations between VPA blood concentrations and multiple clinical variables, including daily dose, age, gender, biochemical parameters (liver and renal function), electrolyte levels, and combination therapy, providing a broader perspective than previous studies that examined only selected factors. It is further strengthened by its specific focus on pediatric patients with epilepsy, an often underrecognized population with ongoing developmental changes and substantial pharmacokinetic variability. These features enhance the clinical relevance of our findings for individualized VPA therapy and TDM in pediatric practice. Despite these strengths, several limitations should be acknowledged. First, this retrospective study is suitable for identifying associations between clinical variables and VPA blood concentrations, but it cannot support causal inference or evaluate within-patient changes over time. Second, daily dose per kilogram of body weight could not be consistently calculated because body weight data were either missing or not recorded contemporaneously in this cohort; therefore, dosage was analyzed primarily on the basis of absolute daily dose to reduce the risk of selection bias.

## 5 Conclusion

This study comprehensively evaluated multiple clinical factors influencing VPA blood concentrations in pediatric patients with epilepsy. The results demonstrated a positive correlation between daily dose and VPA blood concentrations; however, substantial inter-individual variability in VPA blood concentrations was observed, particularly at higher dosing levels. Age represented a major determinant of VPA blood concentrations, and younger children (<6 years) showed lower VPA blood concentrations and received lower daily doses. Among the liver and renal function indicators, creatinine and globulin were positively associated with VPA blood concentrations, whereas alanine aminotransferase was negatively associated with VPA blood concentrations. Moreover, elevated calcium and phosphorus levels were independently associated with reduced VPA blood concentrations. Among patients receiving VPA in combination with only one additional antiepileptic drug, the type of the additional drug had no significant impact on VPA blood concentrations. Collectively, these findings underscore the complexity of VPA pharmacokinetics in pediatric patients with epilepsy and the necessity of individualized dosing strategies.

In conclusion, TDM plays a critical role in optimizing VPA therapy in pediatric patients with epilepsy. By integrating age, organ function, electrolyte levels, and concomitant medications into dosing decisions, clinicians can more accurately achieve and maintain therapeutic concentrations. Routine monitoring and personalized adjustment based on these variables may enhance seizure control, reduce the risk of adverse effects, and improve overall treatment outcomes in this vulnerable population. This was a retrospective study conducted at a single center, which may limit the generalizability of our findings. Future prospective studies with larger, multicenter cohorts and longer follow-up periods are warranted to validate these findings and further explore the factors contributing to variability in VPA blood concentrations among pediatric patients with epilepsy.

## Data Availability

The raw data supporting the conclusions of this article will be made available by the corresponding author upon reasonable request.
